# Adventitial dissection with advanced vessel-sealing for carotid body paraganglioma: A 2-year recurrence-free case report and technical note

**DOI:** 10.1016/j.ijscr.2025.111950

**Published:** 2025-09-18

**Authors:** Werda Majd, Amouri Salim, Chaabouni Mohamed Amine, Medhioub Fatma, Charfeddine Ilheme

**Affiliations:** a ENT – Head and Neck Department, Habib Bourguiba University Hospital, Sfax, Tunisia; b Department of Anesthesiology and Intensive Care, Habib Bourguiba University Hospital, Sfax, Tunisia; c Department of Intensive Care of Mahres Hospital, University of Medicine, Sfax, Tunisia

**Keywords:** Carotid body tumor, Subadventitial dissection, Vessel-sealing, Shamblin classification, Paraganglioma surgery

## Abstract

**Introduction and importance:**

Carotid body paragangliomas present unique surgical challenges due to their hypervascular nature and proximity to critical neurovascular structures. Subadventitial dissection combined with vessel-sealing techniques represents a technical option to achieve complete resection while minimizing complications, though long-term outcome reports remain limited.

**Case presentation:**

A 52-year-old male presented with a 2-month history of a pulsatile left cervical mass associated with chronic neck pain. Examination revealed classic clinical signs (Kocher's and Fontaine's positive). Imaging demonstrated a 3.5 cm Shamblin I tumor at the carotid bifurcation with with <180° arterial contact (Arya I) and preserved vascular anatomy. Biochemical testing confirmed a non-secreting profile. The patient underwent en bloc resection via subadventitial dissection and vessel-sealing, achieving complete tumor removal without vascular reconstruction or nerve injury.

**Clinical discussion:**

This case highlights several key technical considerations in carotid body tumor management. The subadventitial plane provides a dissection barrier, facilitating hemostasis and vascular preservation. Vessel-sealing devices may complement traditional techniques for vascular control. The 2-year recurrence-free outcome supports this approach for selected tumors, though longer follow-up is needed. Cranial nerve preservation highlights the importance of meticulous dissection.

**Conclusion:**

Subadventitial dissection with vessel-sealing can achieve favorable outcomes in Shamblin I carotid body paragangliomas, enabling complete resection with neurovascular preservation. Further evaluation in larger cohorts is warranted.

## Highlights

• Technical Approach: Adventitial dissection with vessel-sealing technology for carotid body paraganglioma resection.
• Outcomes: Minimal blood loss and 2-year recurrence-free survival, supporting surgical management of selected tumors.
• Educational Focus: Illustrates neurovascular preservation (hypoglossal/vagus nerves) with intraoperative documentation.

## 1 Introduction

Carotid body paragangliomas (CBPs) are rare neuroendocrine tumors arising at the carotid bifurcation, with an estimated incidence of 1:30,000 [1]. While typically benign, their management remains surgically challenging due to rich vascularity and proximity to critical neurovascular structures [2,3]. The Shamblin classification predicts surgical complexity, with Type I tumors (as in our case) demonstrating limited arterial contact (<180° per Arya classification) requiring meticulous dissection [4,5].

Traditional approaches emphasize subadventitial resection to minimize vascular injury [6]. Energy-based vessel-sealing devices may complement conventional techniques for hemostasis [7], though comparative evidence remains limited. Complete resection remains the gold standard [8], with cranial nerve injury rates reported in 15–30 % of cases [1].

Non-secreting CBPs (85–90 % of cases [1]) permit elective surgical planning, unlike functional tumors requiring preoperative alpha-blockade [3]. This case illustrates how subadventitial dissection with vessel-sealing can address surgical challenges while preserving neurovascular structures—a strategy supported by technical reports [2,6] but requiring further long-term validation [3,5].

Informed consent was obtained for publication. No funding was received. The authors declare no conflicts of interest. This case report has been reported in line with the SCARE checklist [9].

## 2 Case presentation

A 52-year-old man with no significant medical history presented with a two-month history of a slowly enlarging, pulsatile left cervical mass accompanied by chronic neck discomfort. Examination revealed a firm, non-tender 3.5 cm mass demonstrating classic clinical signs including positive Kocher's maneuver (reduced mobility with carotid compression), Fontaine's sign (vertical fixation), and the Reclus-Chevassu phenomenon (transient shrinkage upon proximal carotid compression). The mass exhibited horizontal mobility but remained fixed vertically, with auscultation revealing a continuous bruit but no palpable thrill.

**Fig. 1:**
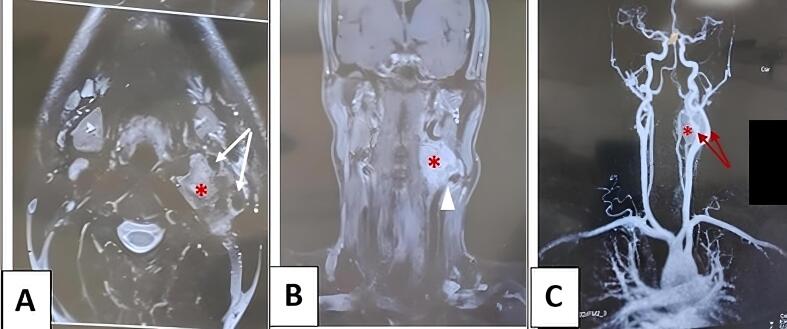
Cervicofacial magnectic resonance angiography. A: Axial view demonstrating an intensely enhancing mass (asterisk) in direct contact with both carotid arteries (white arrows). B: Coronal view showing the same mass (asterisk) exerting mass effect on the carotid artery, which is displaced laterally (white triangle). C: Vascular phase coronal reconstruction confirming preserved luminal integrity of the carotid arteries (red arrows) with a clear cleavage plane between the vessels and mass (asterisk). (For interpretation of the references to colour in this figure legend, the reader is referred to the web version of this article.)

Preoperative Magnetic resonance angiography (MRA) with contrast enhancement demonstrated a well-circumscribed, hypervascular mass measuring 3.5 × 2.8 cm at the carotid bifurcation, showing characteristic splaying of the internal and external carotid arteries with less than 180° circumferential contact (Arya I classification) and preserved vascular luminal integrity (Fig. 1).

Comprehensive biochemical evaluation confirmed a non-functional tumor profile with plasma metanephrines (0.45 nmol/L) and normetanephrines (0.60 nmol/L) well within normal limits, along with unremarkable chromogranin A and catecholamine levels.

**Fig. 2:**
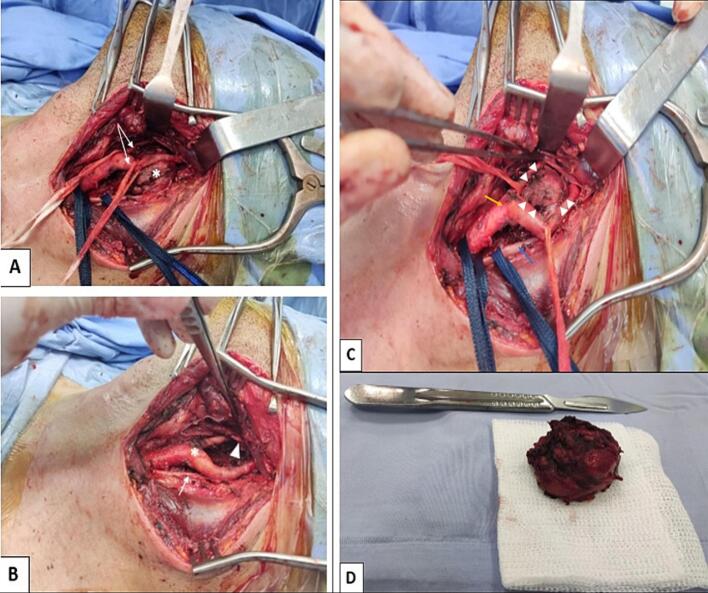
Intraoperative photographs demonstrating: A: Dissection of the tumor (asterisk) with preservation of the carotid axis (white arrows). B: Surgical field after tumor resection showing intact carotid bifurcation (asterisk), vagus nerve (white arrow), and hypoglossal nerve (white triangle).

Surgical intervention was performed through a standard vertical cervical incision along the anterior border of the sternocleidomastoid muscle. Meticulous dissection identified and preserved all critical neurovascular structures, including the hypoglossal nerve coursing superior to the bifurcation and the vagus nerve positioned posteriorly, both carefully protected without requiring intraoperative neuromonitoring (Fig. 2).

C: Critical technical view - Close-up of subadventitial plane (white arrowheads) between tumor pseudocapsule and carotid artery (yellow arrow), with vagus nerve (blue arrow) protected posteriorly.

D: Excised tumor specimen demonstrating intact pseudocapsule.

The tumor was systematically dissected using precise subadventitial technique, developing the natural avascular plane between the lesion and carotid sheath using microsurgical instruments complemented by modern vessel-sealing technology for optimal hemostatic control. This approach facilitated complete en bloc resection without vascular injury or the need for reconstruction, achieving excellent hemostasis throughout the procedure. The surgical field remained exceptionally clean with negligible blood loss, though no specific volume measurement was recorded due to the minimal suction requirements (Fig. 2).

The patient experienced an uncomplicated postoperative recovery with no neurological deficits or vascular complications. Rigorous follow-up surveillance including contrast-enhanced MRI studies at 6, 12, and 24 months demonstrated no evidence of residual or recurrent disease. Clinical examinations consistently confirmed preserved cranial nerve function and absence of vascular abnormalities. At the two-year follow-up mark, the patient remained completely asymptomatic with no signs of recurrence, maintaining excellent quality of life and normal cervical function. This outcome supports the technical feasibility and mid-term efficacy of the described surgical approach for carefully selected carotid body tumors.

## 3 Discussion

The present case demonstrates successful management of a carotid body paraganglioma using subadventitial dissection [2,6] combined with vessel-sealing technology, achieving complete tumor resection while preserving neurovascular integrity. The observed hemostasis compared favorably with historical reports documenting 200–800 mL blood loss in conventional resections [5,7], suggesting potential advantages in blood conservation. Our approach aligns with current literature emphasizing subadventitial dissection for tumors with <180° arterial contact (Arya Type I) [2,4], which may optimize carotid preservation and reduce cranial nerve morbidity. The two-year recurrence-free outcome supports early surgical intervention for moderate-sized lesions [4,8], before progression to more complex anatomical relationships occurs.

The case challenges conventional paradigms regarding preoperative embolization [7,10], suggesting that modern vessel-sealing techniques may offer comparable vascular control for selected tumors without embolization-associated risks [5,7]. This proves particularly relevant for non-secreting tumors [11] where surgical timing remains elective. Our experience reinforces cranial nerve preservation as the critical quality metric [[2], [3], [4]], with methodical nerve protection mirroring contemporary multidisciplinary approaches that show 8–12 % nerve injury rates versus 20–30 % historically [2,6].

Several technical insights emerged: The avascular plane between tumor pseudocapsule and carotid adventitia [6,12] permitted controlled dissection. Vessel-sealing facilitated feeder vessel control without traditional ligation [4,5]. Notably, carotid reconstruction proved unnecessary despite the tumor's proximity, attributable to precise subadventitial technique [2,6]. These observations suggest technological advancements may expand surgical options for selected cases.

Important limitations include the single-case nature and intermediate follow-up [3]. While 90 % of recurrences manifest within four years [3,11], current standards mandate lifelong surveillance [11]. The absence of comparative data precludes definitive conclusions about vessel-sealing advantages [4,5,7], highlighting the need for controlled studies. Technical reproducibility requires validation across different teams [2,13].

This experience supports subadventitial dissection with vessel-sealing [2,6] as a viable option, balancing oncologic completeness with functional preservation. The approach warrants consideration within modern surgical strategies while awaiting confirmation through larger series with extended follow-up [2,3]. The case underscores the evolving paradigm where precision techniques supplant radical resection when margins appear uncertain [2,4,6].

## 4 Conclusion

This case demonstrates that subadventitial dissection combined with advanced vessel-sealing technology is a highly effective surgical strategy for managing Arya Type I carotid body paragangliomas. The technique successfully addresses the primary challenges of this surgery—profuse tumor vascularity and the risk of neurovascular injury—by facilitating a precise dissection that ensures complete resection while preserving the integrity of the carotid artery and adjacent cranial nerves. The excellent functional outcome and absence of recurrence at two years support the adoption of this approach for eligible patients, particularly those with non-secreting lesions amenable to elective intervention. While validation through larger, long-term studies is warranted, our experience confirms that this method represents a significant technical advancement aligned with the modern principles of functional and safe surgery.

## Author contribution

1. **Werda Majd**: Conceptualization, Data curation, Formal analysis, Investigation, Methodology, Project administration, Resources, Supervision, Validation, Visualization, Writing – original draft, Writing – review & editing.
2. **Amouri Salim**: Investigation (anesthetic management), Resources, Writing – review & editing.
3. **Chaabouni Mohamed Amine**: Investigation (surgical assistance), Resources, Visualization, Writing – review & editing.
4. **Medhioub Fatma**: Data curation (postoperative care), Resources, Writing – review & editing.
5. **Charfeddine Ilheme**: Investigation (surgical team), Resources, Supervision, Writing – review & editing.

**Corresponding author**: Werda Majd – Oversaw all stages of manuscript preparation and submission.

## Consent

Written informed consent was obtained from the patient for the publication of this case report and accompanying images.

## Ethical approval

Ethical approval was not required for this case report, as per institutional guidelines. However, all ethical principles were adhered to in the preparation of this manuscript.

## Guarantor

Werda Majd.

## Research registration number

This case report does not require a registration.

## Funding

This research did not receive any specific grant from funding agencies in the public, commercial, or not-for-profit sectors.

## Conflict of interest statement

The authors declare that they have no conflicts of interest related to this work.
